# The instantiation of values

**DOI:** 10.1186/2193-1801-2-166

**Published:** 2013-04-16

**Authors:** Zoltan Balazs

**Affiliations:** Institute for Political Science, Corvinus University, Budapest, Budapest, Hungary

**Keywords:** Instantiation, Value, Love, Envy, Compassion, Malicious pleasure

## Abstract

The paper argues that values are universals, either properties or relations. For the latter, instantiation is a matter of the content of the relation, the relata, and the context (the instantiation of further relations). This moderate realist conception helps us solve some notorious problems in axiology, i.e. malicious pleasure, compassion, envy, and perverted love. Another other important implication of this conception is that there is a further property, the one of being a value. It is a higher-order property which can be instantiated by relation (and property) values to various degrees. Finally, the questions of whether particularity is a value and how rarity can be a value are also addressed. The underlying ontological conception provides a sound way of finding a answer to these questions as well.

## Instantiation: connecting universals to particulars

For axiology, problems such as wicked or perverse love, malicious pleasure, suffering because of another person’s pleasure, or compassion have divided theorists. What seem to be unconditional values (love, pleasure) may become part of something bad; what seems to be an unconditional disvalue (suffering) may become part something good (suffering because of the other’s suffering). Values and disvalues appear to form complexes or wholes whose axiological index may be different from that of its components. But how is this possible? Simply declaring that there can be such a difference is not an explanation. A more convincing answer is needed. The present attempt relies on a realist account of values and directs the focus on instantiation which is considered responsible for these differences.

In a realist account of values, values are universals. Beauty is a value that can be predicated of various particulars. Politeness is another value, predicable of various particulars. Love is also a value, but it presupposes at least two particulars. Similarly for trust or forgiveness. We can thus distinguish between property values and relation values. Some values are less unequivocally categorizable: pleasure might be an example. It is possible that we simply ‘feel’ pleasure without there being an object to which our pleasure is related, but in most, perhaps virtually in all, cases we are ‘pleased at’ something, hence probably the ontologically proper rendering of pleasure is always ‘being pleased at,’ which is a relation. Similarly,’ love’ is more properly rendered as ‘being in love of’ or ‘loves’ or ‘being loved by.’

It seems that there are disvalues, too, though axiological literature seldom mentions the concept of disvalue^1^, not to speak of *intrinsic* disvalue. Our axiological vocabulary is, however, full of concepts denoting properties and relations with a negative axiological index. Disvalues are not simply missing values, not even in the case when a value’s presence is or was expected (the dinner party was *not* enjoyable, but it was not boring, either), though, of course, disappointment in this case is a disvalue. Nor are disvalues simply ‘deformed’ or ‘misplaced’ values, like the heroism of Don Quijote, since ‘deformation’ or ‘misplacement’ and similar notions refer to (higher-order) relations between values, not to values themselves. The source of such confusions is partly language itself. Some disvalues have their proper names (ugliness, hatred, pain) some are denials or opposites of values (impoliteness). Notwithstanding the pluralism of expressions, the basic idea is alway the same. There are properties and relations we consider, ceteris paribus, not only unworthy of protection and preservation, but often worthy of destruction.

If values are universals, then a number of ontological presuppositions are virtually accepted and the related problems necessarily appear. First of all, the idea that there are universals entails that there are particulars, and the problem of how the two entities are related ensues. The instantiation of universals is one of the greatest problems of realist ontology. In simple words, those who accept the distinction between particulars and universals, need to explain how the latter get instantiated by the former entities. Most realists today reject the Platonic view that ideas (universals) may exist separately from particulars. Their typical view is that only instantiated universals exist. All we can know about particulars are expressed by universals, particulars themselves (in a ‘bare’ state) are inaccessible. Still, they must be presupposed, otherwise the plurality of things would collapse into a single entity. But then it is a problem of how universals get instantiated by particulars.

By going further along this reasoning one may conclude that only particulars exist. This would be the nominalist position. But then it will be necessary to explain why certain particulars are similar in certain respects. Even if complete identity between such ‘respects’ is excluded, that is, the denial of universals is maintained, and only ‘resemblance’ is allowed for, the relation of ‘resemblance’ will be a new ontic entity, obviously not a particular. The problem of how ‘resemblance’ obtains or gets instantiated, reemerges.

Remaining within the realist framework, the problem of instantiation, i.e. how to explain the connection between, or connectedness of, particulars and universals, has been approached in various ways. The focus of these proposals has been largely the ‘tie’ or ‘nexus’ *which* does the job of connecting the two types of entities. Such proposals usually provoke the objection that, nilly-willy, another type of entity is presupposed which is neither a universal, nor a particular. Defenders of such proposals try to fend off this objection by diverting the ontological image of a ‘thing’ that exists out there in reality to a different kind of image, something that is real yet formal. Terms such as ‘non-relational tie’ or ‘aspect’ or ‘sense’ may be used^2^. These attempts will not be discussed here in detail, for such a discussion belongs in an ontological paper. The basic idea all these theorists share and defend might be captured by a very pedestrian example. If I am stopped on the threshold to the house, with one leg in, the other out, I may be said to be both *in* the house and *out of* it, though the two spaces are different, and I am the same person. It is both true that I cannot be at two different places at the same time, and that I am both in and out. What is needed to make sense of this state of affairs is not to abandon the idea of ‘space’ or ‘place’ and the unity of the person, but to introduce a further concept, such as that of the ‘sense’ or ‘respect’. Hence, instantiation is a concept that works with and in its own terms, affecting the reality neither of particulars nor of universals, yet it is just meant to answer the question of how they are connected to each other. In this context, the instantiation of values as universals is merely an applied case of the instantiation of universals and there is no need to discuss it separately.

The problem of how some universals instantiate the supposedly higher-order universal of being a value, is insertable to such a conception, too. For instance, universals such as beauty or politeness are usually considered values, whereas redness or being a cube are not. But there also are many universals that look ambiguous. Think of originality, novelty, awesomeness, prestige, provocativeness, or curiosity. They are surely values to some extent, or in some sense, but not as unambiguously or thoroughly as beauty or love. If such graduality is possible among value universals, then the implication is that being a value is a higher-order property of various universals that is instantiated to various degrees by various universals. Beauty is a value-property all the way through, it may called a pure value universal. Novelty is a value property, or probably a value relation, which is not a pure value universal, since only in virtue of the novelty of something we do not usually attach great value to it, but it does confer some value to most things. Such universals seem to instantiate the universal of being a value to a lesser extent. They are not only values, so it appears. Nevertheless, the instantiation of being a value by another universal is to be explained in terms of instantiation^3^.

## How do universals obtain?

Instantiation is, therefore, an ontological operation or event. But in order to be able to answer the original question, that is, how to account for axiological differences in various value complexes, the notion of instantiation must be extended. The next question is ‘what makes property A obtain?’ Alternatively, ‘in virtue of what is property A instantiated?’ A particular face may be beautiful or ugly, a gesture polite or impolite, a relationship that of love or hatred, and a person may be filled with pleasure or suffering. What we want to know is not merely in how the connection between a face and beauty is established. We want to know in virtue of what the face is beautiful. Of course, there are *natural causes* of a state of affair ‘that a face is beautiful, ‘or’ that Romeo loves Juliet.’ Sciences and arts are there just to discover and explain these causes. Our interest is ontological: given the world of universals and particulars, and on the presumption that their connectedness is explained in terms of instantiation, we still want to know how a particular connection comes about. The problem of instantiation is not solved by examining the tie between universals and particulars, it can be extended to looking for an explanation of how, in virtue of what, such ties or connections there can be.

This question and problem is, again, not restricted to values but here only the instantiation, the coming about of values, will be discussed. If the results are sound, then they may be generalized to non-value universals, too. But before the discussion of concrete examples begins, let me briefly refer to a related topic. This is the problem of value-bearing. The usual question asked is ‘*what* things/entities bear value?’ On the conception of instantiation outlined so far, another question to be asked is ‘*how* do things/entities bear value?’ Thereby the relation of ‘bearing’ or ‘bears’ is replaced by the relation of instantiation. This is a simple terminological operation. Given the prevalence of the first question, however, it is necessary to reflect on it briefly^4^.

Let us say a face is beautiful. Then it is a particular that bears *the* value *of* beauty. Alternatively, we may say that the face is beautiful yet it is the one millionth reproduction of a Raffaello Madonna, and conclude that ‘it’ is not (really) valuable. ‘It’ refers here to the copy, of whatever form, and entails that beauty is just one aspect of it that needs to be taken into account, yet ‘its’ value is not ‘born’ by beauty only. Other instances of value bearing may be even more complicated. Particulars are usually interpreted as persons or objects, but value judgements are often made of experiences and events. If I recall a pleasant experience, I may feel actual pleasure, and conclude that certain memories are pleasurable or pleasing (and others painful). An inauguration ceremony may be dignified and hence valuable. Those whose ontology is based on tropes and does not allow for particulars and universals as separate entities will assert that it is tropes that bear values. Wittgensteinians say that the world contains only facts or states of affairs, wherefore values are ‘born’ by facts or states of affairs. It is a value *that* the car is beautiful or *that* I love my child. On such an account, beauty and love are not themselves values but value-making qualities or features, like in the first example of the copy of the Raffaello painting.

It appears thus that the answer to the question of ‘what bears value’ depends on one’s ontological preconception. In a sense this is indeed the case. If there are no particulars/universals, *they* cannot bear values. If the world is made of states of affairs, only states of affairs can bear value. But the issue is not so simple. The notion of ‘value bearing’ itself presupposes a distinction between two kinds of entities: those that have a special relation (that of bearing) to another type of entity, value(s). Thus, it appears that those who find the question ‘what bears value?’ meaningful already make an implicit distinction between ‘value-bearers’ and ‘value(s)’. The brackets are important. For the next question is whether that which is born by ‘value-bearers’ is simply ‘value’ or it can be several ‘values.’ The literature on intrinsic value, ever since Moore introduced this term in his *Principia Ethica*^5^, has been inclined towards the interpretation of the problem of value bearing in terms of ‘intrinsic value being born by’ this or that. However, nothing compels us to follow Moore here. Not only intrinsic value, but any kind of value may belong to the set of entities that are related to another set of entities by the relation of ‘bearing.’ What remains inevitable is the precommitment to the view that values or (intrinsic) value are entities different from those that bear them. But this is, of course, the realist view.

But if ontological realism is accepted, then the question of where value is to be found will be answered by saying: among universals. It is possible that we shall have to make a further distinction between values and intrinsic value, but the relation of ‘value bearing’ will first appear between particulars (whether they are persons, objects, or events) and universals, that is, concrete values. Even if the one millionth copy of the Raffaello Madonna is much less valuable than the original painting, beauty is still born by each copy *and* the original. It is just that further values are born by the original which make it more valuable. It is also possible that some special value arises out of the complex whole, or bundle of value properties, which the original possesses, but not the copies (more on this example later). On this Moore’s intuition was probably right. But this is just when the other question, namely, ‘how are values born?’ or ‘how do things bear value(s)?’ becomes urgent and interesting.

In what follows I shall consider particular cases and make suggestions of how to any value bearer might be analysed and the instantiation of values be explained. There won’t emerge a single winning way of explaining the instantiation of values, from which it follows that instantiation is an ontological phenomenon that may have various causes, some being active and others passive here but not there and vice versa.

## Perverted love

Compare Hitler’s love of Germany and Churchill’s love of England. We surely want to make a sharp distinction between the two cases in terms of value. How can we make it?

One possibility is to take the two cases ‘organic wholes’ in the Moorean sense and simply assert that the first case does not represent ‘intrinsic’ value (or much less), whereas the second case does (or much more). But this is insufficient. An explanation is required, since the structure of both ‘wholes’ is identical ([*h*L*g*] and [*c*L*e*]) and the universal of Love which is a relation value, henceforth rendered as ‘Loves,’ occurs in both wholes. There are three logical possibilities. First, that ‘Loves’ is not instantiated, after all. It is a mistake to think it is. Second, that the particulars involved instantiate further values and disvalues which account for the difference of the two cases. And third, that despite the apparent similarity, the ‘structures’ of the two cases are different. Let us examine each possibility.

*First*, suppose that the content of the relation ‘Loves’ is indeed the same in both cases. Both Hitler’s love of Germany and Churchill’s love of England involve the required positive affective, intellectual, volitional, etc. aspects of the relation ‘Loves’^6^ and both persons and collectives involved are adequate subjects of love^7^. Thus, there is no ontological difference between them. Given the identity of the content of ‘Loves’ in both cases, it is possible that both Hitler’s love for Germany and Churchill’s love for England are true instances of ‘Loves.’ But we may speculate further and, especially given the complexity of ‘Loves’ as a relation value, find differences. For instance, since Hitler’s love of Germany turned out to be compatible with his willingness to destroy it, we may conclude that he hated Germany at least as much as he loved it, and such an ambivalence is simply incompatible with love. This is another relation, which may lack a linguistic term, yet ontologically different from both love and hatred. Alternatively, we can imagine some second-order relations intervening, such as corruption or deformation, as in the case of Don Quijote’s misguided heroism. Of course, the instantiation of second-order relations raises further questions, but it is sufficient here to allow for the possibility that, for instance, the proposition ‘Hatred Corrupts Love’ is ontically grounded. The result is the same: ‘Loves’ is different in the two cases. It holds for [*c*L*e*] but it does not hold for [*h*L*g*]. Rather, the latter relation is [*h*CL*g*], that is, Hitler ‘pervertedly loves’ Germany, or [*h*HL*g*], that is, Hitler ‘hates/loves’ Germany.

*Secondly*, consider the what seems to be the most plausible option, namely, that it is the two persons who account for the difference between the two cases in terms of value. Unusual as it may sound, we must assume (1) that further values and disvalues being instantiated by Hitler and Churchill contribute to the value of the two wholes, in this example, crucially; and (2) that these values and disvalues are in some sense aggregable, yielding a judgement about the valuableness or worth of each person, by which we are able to account for the axiological difference between the two cases. Thus, even though both persons ‘Love’ their respective countries equally (it is also presupposed that there is not any difference in terms of the intensity or strength of the relation ‘Loves’ in either case, where ‘intensity’ might be another second-order relation), the value of ‘Loves’ contributes to the two persons’ worth or valuableness only marginally. There are other values and disvalues which they instantiate and which may be greater and more significant than these. From this argument it follows that the axiological difference between ‘Hitler loves Germany’ and ‘Churchill loves England’ supervenes on values and disvalues only virtually present in the two propositions^8^. Thus, in the strict sense, we are not explaining the axiological difference in terms of ‘Loves’ but of other value universals, and of the valuableness or worth of the relata.

But it also seems possible to insist on the initial suggestion, namely, that the two cases can be explained in terms of the relation value ‘Loves,’ but with the help of the concept of valuableness or worth. Suppose that the total sum of values and disvalues Hitler instantiates results in a negative worth of him. Notice that it does not follow that he hasn’t got any value whatsoever, and a single value may be thought to be sufficient to justify *some* ethical protection (for instance, against torture as punishment). Still, it is theoretically and axiologically possible to conclude that he possesses negative worth. It may then be suggested that for ‘Loves’ to get instantiated a positive worth is a necessary precondition. It is impossible for certain persons to ‘Love’ anything, despite the emotional, intellectual, even moral, similarities of their affection toward, for instance, their children and the affection other, ‘normal’ people have to theirs. If this sounds absurd, think of the horrible case of the Goebbels who killed their children prior to their own suicide. It makes sense to ask the question: did they ever ‘Love’ their children? (Contrast this case with Salomon’s test of the two women claiming a baby to be its mothers!) Thus, we may say that even though ‘Loves’ is a value, its being instantiated presupposes the instantiation of the second-order property of having positive worth of the *relata*.

To this suggestion it might be objected that it makes relation values *partly* supervene on other values, and thus *partly* second-order relations, which is inconsistent with the general conception of values as first-order universals. However, even if values are first-order universals, their *instantiation* may sometimes depend on other values’ being instantiated. Think of the Platonic concept of justice (or morality, a kind of social and moral harmony) which presupposes the right ordering of other virtues, namely, modesty, courage, and wisdom. Thus, it is possible that the relation value of ‘Loves’ also presupposes other values being instantiated, in this case, positive worth. The result is, again, that Hitler’s love of Germany turns out to be something else than love, since *his* axiological worth is negative. In general, the instantiation of relation values might be blocked if the relata, or at least one of them, have negative (or very low?) worth. For it is also possible that it is the other relatum that blocks the instantiation of the relation value: in E. T. A. Hoffman’s tale, *Der Sandmann*, Nathanael falls in love with Olympia, who/which is a doll; is it a *‘Love’* affair? The puzzling case of Don Quijote is similar, his heroism is, on this account, not heroism at all.

Another objection is that the worth of the particular cannot influence the instantiation of a value (and possibly not only relation, but property values as well), since worth and valuableness themselves supervene on the values that are instantiated. Hitler’s love for Germany as love cannot supervene on Hitler’s worth, since his worth partly supervenes on his love for Germany. This is indeed a logical consequence of the reasoning but not necessarily an argument against its consistency. The structure of the problem is similar to the one of the relationship between our moral actions and moral characters. Character traits follow from our actions, and actions flow out of our character traits. Moral worth is a result of values we have, and whether or not specific values obtain is partly a matter of our moral worth^9^.

There are cases where not only one, but both relata have negative worth. Obviously, in such cases the instantiation of the relation value in question is double-blocked. Thus, trust between burglars is, axiologically (and even perhaps psychologically), not trust – as a value – at all; the devil’s being pleased at destruction is not a value. Of course, it requires a very accurate moral examination to conclude that a person has ‘negative worth’ (things, objects, ideas having negative worth are much easier to argue for), and one may doubt that such a conclusion can ever be justified. This is, however, a problem for ethical theory.

*Thirdly*, we may reinterpret the alternative suggestion that was made in the first point about hate and love being mutually exclusive relations. According to it, if both hate and love can be predicated of an empirical relationship, we have to deal with a relation that is neither love, nor hate, but something else. Our vocabulary (in fact, the vocabulary of a particular language) may or may not contain an independent word for this, and there is an ample room for further psychological and moral analysis of such phenomena^10^. Now the reinterpretation of this possibility takes a different route. It refers to the *context* of the relation.

Think, for instance, of the non-value relations of ‘being left to’ and ‘being right to’. These are essentially context-dependent relations, that is, their instantiation is ontologically not only a matter of the two things being spatially related, but equally a matter of the point of view the relation is predicated from. That point of view implies another relation, and thus we have three relations, even though only one of them is (e.g. ‘*a* is left to *b*’) actually predicated. Relation values may be similar. They may supervene not only on the particulars they connect and on the content of the relation, but also on the presence or absence of other relation values which constitute the contexts for them. It is not easy to tell whether or not ‘Loves’ in our case is in fact instantiated, because it is very much a matter of other value (and disvalue) relations’ being also instantiated. Even if Hitler ‘Loves’ Germany, his hatred of, and despise for, other peoples and nations provides a context for ‘Loves’ that blocks its instantiation. The argument is not that ‘Loves’ and ‘Hates’ both contribute to the worth of the individual, which influences the instantiation of both, since that would be falling back to the previous argument. Instead, it is supposed that certain relation values and disvalues are especially sensitive to some other values and disvalues (mainly to their counterparts) that provide a special context for them. If it is true that ‘*a* is left to *b*’ provided that I occupy a position *c*, it may not be true if I occupied a position *d* (which might be on the other side of the virtual line between *a* and *b*, or exactly on the line, or simply my turning my back to them). Of course, it is not as easy to determine whether ‘Loves’ or ‘Hates’ is in fact instantiated as to determine from a fixed point whether *a* is left to or right to *b*. ‘Loves’ and ‘Hates’ are themselves complex value relations, intertwined with values and disvalues by higher-order relations. It is merely a possible and reasonable assumption that ‘Loves’ and ‘Hates’ provide special contexts for one another that mutually play a constitutive role in their instantiation.

To sum up: Hitler’s love for Germany (1a) may be a true instance of love, but of minor ethical importance; (1b) may be something else than love, as a result of its being combined with his hatred of Germany; (2) may not be instantiated at all, because his axiological worth is negative; (3) may not be instantiated at all, because his hatred (of other particulars) provided a context for love that blocks its instantiation. The general lesson is that the instantiation of values supervenes on the relation (its content), the relata, and the context.

## Malicious pleasure

It will be instructive to see how these results can be applied to the case of malicious pleasure (‘Schadenfreude’) which has been a notorious problem of axiologists ever since Brentano. In a recent discussion of it which draws on Brentano’s, Ross’s and Chisholm’s related views, Noah Lemos concludes that “we may say that the property of *being pleased that someone is suffering* is not an intrinsically good-making property (…) [but] it hardly follows that the property of *being pleased* is not (…). These are, after all, distinct properties, since one can have the latter without having the former. (…) The fact that *someone is pleased* is different from the fact that *someone is pleased that someone is suffering*^11^.”

Now it is hard to understand *how* it can be the case that *being pleased* is an intrinsically good-making property whereas *being pleased that someone is suffering* is not. *That* the two cases are different is clear, *how* their difference is to be accounted for, is unclear. Lemos holds the view that the bearers of intrinsic value are facts, not properties. But his own conclusion makes it clear that the difference between facts must rest on some differences between (good-making) properties, therefore we must seek an answer *within* the facts themselves. His mere asserting that the two properties are different, however, is insufficient.

To begin, it should be noted that neither ‘being pleased,’ nor ‘being pleased that someone is suffering’ are, strictly speaking, properties. Both are expressions of facts, thus, the problem of how to account for a difference between facts is, in the quotation above, simply transposed to the realm of universals. This is why the difference between the two putative properties remains puzzling. Let us, therefore, reformulate the two ‘properties’ as ‘being Pleased at’ and ‘being Pleased at Suffering (of) (at/from)’ (read, for instance,’ Jim is pleased at Joe’s suffering from headache.’) Evidently, these are relational entities, or simply, relations. The first is a simple universal, the second, involving a reference to another relation (‘Suffers at/from’), appears to be a complex universal. For sake of grammatical simplicity, let us replace ‘being Pleased at’ with ‘Enjoys^12^.’

Following the line of argument about the instantiation of the relation value ‘Loves’ it may first be assumed that the content of the relation value ’Enjoys’ is the same in Schadenfreude as in other, morally acceptable, cases of enjoyment. Then it can be maintained that the enjoyment of suffering is a true case of enjoyment, and perhaps add that ethical theory must depart here from axiology^13^. But it seems possible to argue that enjoying suffering or pain is always a perverted or an impure kind of enjoyment, or perhaps a combination of sentiments and feelings that is fundamentally, ontologically different from enjoyment, a case of joy, even though there is no separate word for that^14^. Briefly, if we want to maintain the enjoying suffering is not a true case of enjoyment, thus, the value of ‘Enjoys’ is not instantiated, we may insist that another relation value (if it is a value at all) is instantiated.

Secondly, the instantiation of enjoyment as a value is not merely a matter of the content or the relation but also of the relata and the context. Thus, even if the relation ‘Enjoys’ is instantiated and it relates adequate relata^15^, there is the possibility, outlined above, that certain axiological properties instantiated by the relata influence the instantiation of the value of ‘Enjoys.’ Unlike the complexities of ‘Loves,’ the worth of the relatum that is being enjoyed is usually easier to determine. Pain and suffering are in most cases disvalues, even though they might have some value aspect as well (I might enjoy the [sight of? experience of? a] painful tiredness of my son, after a long bicycle tour). But enjoying disvalues is hardly a value. Thus, in the case of ‘Enjoys the Suffering (of) (at/from),’ the value relation of ‘Enjoys’ is not instantiated, after all.

It is here where the fact that in ‘Enjoys the Suffering (of) (at/from)’ we have to deal with two relations becomes relevant. For suppose that the suffering of a human being is well-deserved. Some scholastics opined that the sight of those suffering in the Hell is a source of enjoyment for those in the Heaven. The idea behind the justification for such an enjoyment is that Hell is a result of divine justice and suffering in it is deserved. If one enjoys deserved suffering, one does not enjoy a disvalue, but something worthy (usually, but not always, due to moral reasons and disvalues)^16^. We may then interpret the case in point such that ‘Enjoys the Suffering of a Wicked person’ where ‘wicked’ may be replaced by ‘having negative worth.’ Of course, to arrive at an ethically right judgement of ‘Enjoys,’ one needs to look deeper in the individual case. It may also be argued that in ‘deserved suffering ‘desert’ and ‘suffering’ must be delicately and minuciously balanced so that the pain or suffering wipes off, as it were, the transgression. This may happen automatically, for instance, when a child feels immediate pain after wounding his finger despite warnings, but often it needs external remedy. In such cases we usually have a number of further requirements which are, of course, not our concern here. It suffices to note here that unless punishment is proportionate to the misdeed, suffering becomes a disvalue and ‘Enjoys’ is not instantiated.

It is also arguable that what gets instantiated in such cases is not ‘Enjoys’ but ‘is morally Satisfied at’ or something like this; or perhaps ‘Enjoys Justice’ which, obviously, would dispel all reservations about enjoying some cases of suffering as being only apparent cases of ‘Enjoys Suffering (of) (at/from).’ The point remains, namely, that a change in the relatum (carrying out a morally evil action, or, repentance), which may partly be brought about by further relations’ being instantiated (the wrongdoer may suffer from a disease wholly unrelated to his misdeeds) affects the instantiation of the relation. Such a change may block it.

Thirdly, since enjoyment and suffering (pleasure and pain) are related to one another in an opposite way, it is a reasonable assumption that they play negatively constitutive, blocking roles in one another’s instantiation. But it is impossible to tell in principle where enjoyment ceases and something else (hatred, despise, cruelty) replaces it; or where ‘pure’ suffering ceases and something else (e.g. courage), replaces it. It is psychological suffering that is especially complicated. Think, for instance, of jokes told at another person’s expense in his presence. His suffering might be undeniable, yet the enjoyment of his pain by others might not be entirely bad. Sometimes suffering of this kind is fundamentally, ontologically, tied up to certain values such as comradeship, equality, which surely are values worthy of enjoyment.

It is hardly necessary to consider in detail further cases of “mixed wholes” or “organic wholes” in which suffering or pain and pleasure or enjoyment participate. The analysis of such cases runs on the same track as in the case of malicious pleasure. Take some complicated cases, such as the following: Jim suffers at Joe’s partly undeserved enjoyment of a reward. Or an even more complicated case: Ann suffers at Barbara’s suffering at Cindy’s deserved pleasure. In simple words, Barbara is envious of Cindy and Ann feels ‘compassion’ for her. Such cases can be changed *ad libitum*: Cindy might suffer at Barbara’s being envious of her and Ann may be compassionate with Cindy. Remaining with the last case, we have to do with three persons (particulars) and four relations. (i) ‘Cindy (deservedly) Enjoys something, ‘ (ii) ‘Barbara Suffers at Cindy’s enjoyment, ‘ (iii) ‘Cindy Suffers at Barbara’s envy, ‘ (iv) ‘Ann is Compassionate with Cindy(’s suffering).’ Subscribers to the Moorean conception of intrinsic value rooted in organic unities simply declare what normal moral intuitions tell us, namely, that certain propositions designate facts (‘wholes’) that have different values. If pressed, they might say that as newer and newer relations are added, the ‘value’ of the ‘whole’ changes. Thus, (i) is valuable, (ii) is not, (iii) is valuable (perhaps) and (iv) is also valuable. But in virtue of what these changes occur, remains obscure.

If values are universals, either properties or relations, then we have a conception by which the differences between (i) through (iv) can be explained. It is possible that a value is instantiated in one case but not in another. The ontological grounds for this can be different. As in the former example, it may be the content of the value, it may be the relata (in case of relation values) or the context that is responsible for the instantiation of the value or for the blocking of it.

## Envy and compassion

Enjoyment of what is good and valuable is undoubtedly a value. Enjoying suffering can be interpreted in various ways, and the moral intuition, that in some cases it is fundamentally evil despite the presence of ‘Enjoys’ or something similar to it, can be axiologically justified. Now though the general lessons drawn from the analyses of love and malicious pleasure entail the explanations of further axiologically disturbing cases, such as suffering at another person’s deserved enjoyment which is intuitively not a disvalue, despite the presence of suffering; and that of suffering at another person’s undeserved suffering which is intuitively a value, despite the presence of suffering, it will be useful to outline these explanations.

In the case of being pained by another person’s deserved enjoyment we are inclined to say that it is not only the pain or suffering in itself that is bad, it is *made worse by a value* (namely, the value of enjoyment on the part of the other person). If we find this analysis unacceptable or simply puzzling, we can turn to the usual reasoning, and say that (1a) this is real suffering, though to be morally condemned; (1b) this is not real suffering, despite the emotional, psychological, etc. similarities, but something ontologically different for which we have, in this case, fortunately, a separate word, envy^17^; (2) this is not real pain or suffering because a disvalue presupposes the negative worth of the particular at which the pain or suffering is directed, which does not obtain here^18^; (3) as said above, enjoyment and suffering are counterparts, being specially related to one another, and the deserved enjoyment of the other person blocks the instantiation of suffering as a real disvalue.

Compassion (shared suffering, *Mitleid*) is generally considered morally praiseworthy. In practice, much caution is required, however. Hitler’s anguish about losing the war does not justify compassion. But being compassionate with someone undeservedly suffering is a value. Again, on the face of it, compassion seems to be a value based on disvalue, or on two instances of the disvalue ‘Suffers.’ (1a) One may insist, therefore, that compassion is suffering, axiologically a disvalue, yet ethically right. (1b) Alternatively, compassion may be something different from suffering, pain, not with standing certain similar features^19^. (2) As enjoying a disvalue is no more a value but a disvalue, suffering because of a disvalue is a value. In the first case, enjoyment loses its value aspect, that is, it is no more a relation value. In the second case, suffering, which is overwhelmingly, but not entirely (see the example of tiredness), a disvalue, takes on a value aspect, and becomes a relation value. If the suffering of the other person is deserved, compassion may take on a disvalue aspect, and become a relation disvalue (sentimentalism, perhaps). Notice that in these cases we are talking not only about the instantiation of a value or a disvalue, but also of the universal ‘being a value.’ (3) Finally, it could be argued that compassion is strongly connected with love, even in the case of strangers^20^, and it is love that ‘helps’ compassion as a relation value obtain. Even if compassion were always related to love, and no case of being compassionate without love obtained, the two relations were still different, since love does not ‘require’ a disvalue (suffering) to get instantiated. Yet it is possible that love does‘require’ compassion to become more itself, and we have a chain of values in which the instantiation of a disvalue (suffering) plays a certain role which is, perhaps, not simply ‘illumination’ but a kind of constitution. The ethical consequences are, again, up for further discussions.

Compassion may be generalized, as it is a particular case of a value ostensibly ensuing from two disvalues. On a more general level we get that hating evil (being pained by pain, angered by cruelty, resenting injustice, etc.) is good, even though hate and evil are both disvalues. But such generalizations are not always done with sufficient care. It is arguable that the hatred of evil is different from the kind of hatred that is inherently bad. Psychologically, these attitudes are not easy to separate, and may often overlap, which has been a source of much fanaticism in history, but phenomenologically they are not identical. But we do not need to turn to phenomenology and moral psychology to explain the difference. Axiology has its own resources for that. If something exists that rightly deserves hatred, hatred as a disvalue is instantiated, but takes on a value aspect and becomes partly a relation value. The relatum plays a constitutive role not only in the instantiation of a disvalue, hatred, but also in the instantiation of a higher-order property of being a value. And it may be argued that hating evil as a value presupposes the love (and knowledge) of good, an evident value, and thus its instantiation is a matter of the obtaining of certain other axiological facts^21^.

## The instantiation of being a value

The analyses of compassion and hating evil revealed that the instantiation of values is not only a matter of the relation, the relata, and the context, but also of the higher-order property of being a value. The property of being a value is a second-order property, instantiable by first-order properties and relations. As was suggested, there are properties and relations that may instantiate the property of being a value to various degrees or intensity. Moreover, certain properties and relations may instantiate both the property of being a value and of being a disvalue. Thus, it is possible to argue that whereas love is a pure value, enjoyment is perhaps not entirely such; pain and suffering are overwhelmingly disvalues, but may take on value aspects as well^22^. More ambiguous value properties and relations include ‘originality, ‘provocativeness’ as well as ‘competes with’ or ‘is excited by^23^.’ The ambiguity results from the ontological fact that the property of being a value/disvalue does not fully saturate, so to speak, the respective properties or relations.

Exploiting this ontological fact we may find an axiological answer to the disturbing question of what makes otherwise detestable, horrible things, such as the crematoria of Ausschwitz, worthy of preservation; or what makes the Cross worthy of replication and cherishing. Could we say that we preserve the barracks and other remains of Ausschwitz because they are valuable?

In a sense, nothing could be more worthy of destruction. But it is possible to try to make sense of objects of this kind possessing some kind of a value. For instance, we may argue that despite the disvalues such objects possess (usually relation disvalues, such as ‘causing pain’), innocent blood, so to speak, consecrated the instruments of torture for good and they have become ‘holy.’ To clarify the axiological-ontological background of such a value would require a separate discussion. It is merely a suggestion that there is such a value as secular holiness that can arise from human suffering or from any major human achievement, and is instantiated by physical objects. Holiness is one of the purest values, and is probably the integrative value of all religious (religiously relevant) values, working in quite the same way: a special divine presence is it that consecrates a certain thing, place, or time (but also persons, events). If we do feel that there are instruments, places, buildings that ‘demand’ from us a very special attitude, not reducible to other, perhaps similar, attitudes^24^, we may perhaps sense a presence of a special value, that of secular holiness^25^.

But there is another, perhaps more commonsense, argument for not destroying the instruments of utter destruction. This is that those instruments (in the broad sense) *remind of* people of horrible sins committed against innocent people and mankind in general. And this is important and good. But ‘Reminds’ is a relation that can hardly be called a unequivocal or pure value. In many cases we use things to remind us of other people we want to continue hating. Or want to take revenge. In fact, nothing precludes that certain people take inspiration from the sight of Ausschwitz to carry out similar crimes, or, to be more modest, that they get disillusioned by the moral prospects of mankind and become cynics; or that they simply begin to despise the victims for not having resorted to violent resistance against the horrors, and so on. The possible effects of ‘reminds of something disvaluable/worthless’ are by no means unequivocally or necessarily positive. Whether or not they are positive depends usually on the character, and, axiologically, the worth of those affected. If the relation in question participates in the property of value at all, the value, and through it, the property of being a value, is instantiated only if the affected particular (the person reminded) displays the expected positive reactions. The person’s worth plays here a decisive role not only in the instantiation of a relation value, but also in instantiation of the property of value, too^26^.

## Particularity and value

In many cases we feel that the uniqueness or irreplaceability of the particular that instantiates various values as well as the supervening property of being a value confers a special value to it. This is a challenge to the explanation given here for the instantiation of values, since if some value supervenes on a particular’s being unique then not all values are universals. This would shake the grounds of the present conception which presupposes that all values are universals (and that there are higher order universals, including the one of being a value). Let us face this challenge.

The proposition that “*a* has a unique/special value” can be interpreted in various ways. It may mean that *a* has at least one value which makes it prima facie worthy of a positive attitude. Evidently, this interpretation is not related to the problem of particularity. It is merely a semantic tool to emphasize the importance of the respective value that is instantiated.

The proposition may be interpreted in ontologically more meaningful ways. For instance, it may mean that *a* possesses a special combination of values, not possessed by any other particular. But this may be only an existential-practical proposition. As long as the actual or potential existence of another particular, displaying the same combination of values, cannot be excluded, the particular combination of the affected values is repeatable and uniqueness is, ontologically, gone. Suppose, however, that any such possibilities can be excluded. For instance, it is hard to imagine another Mona Lisa, painted by Leonardo as a replica of the one we know, to exist somewhere. Does the one in the Louvre, then, possess a special value, in addition to the aesthetic, intellectual, etc. ones it has? There are, it seems, two possible interpretations of such a particular’s having something unique about it.

First, the special combination or ‘bundle’ of individual values, their interrelations, and the various intensities of their being values are still, in principle, repeatable universals. It is possible to make a copy of the Mona Lisa that is identical with the one painted by Leonardo, except that the new one is not painted by him. The aesthetic enjoyment one draws from contemplating the painting is not affected by the fact that it is the original or a replica. But even if we consider it (or any other complicated particular) practically impossible to copy, uniqueness still attaches to the values, to universals instantiated by the particular painting rather than to the painting as a particular. Perhaps we may speculate further and suggest that there is a higher-order universal (a relation) which may be called ‘being combined uniquely’ but it would be hard to see how the higher-order universal of being a value can be linked to other higher-order universals such as ‘being combined uniquely.’ Further, this suggestion does not dispel the doubts that the very same universals may be ‘combined uniquely’ repeatedly, and the value attached to particularity gets lost, after all. Thus, the supposed higher-order universal of ‘being combined uniquely’ appears to be rather vacuous^27^.

There is, however, a second interpretation of uniqueness which makes an explicit reference to particulars. As it was hinted at above, we usually attribute a special significance to originality, to the fact that a particular artifact has a unique relation to its maker (another particular). The Mona Lisa is unrepeatable because its painter died long ago^28^. Whereas the relation ‘being painted by’ is a universal, the relation ‘being painted by Leonardo da Vinci’ is not, as it essentially refers to a particular. Particulars are unique by definition, and if they enter a relation in an essential sense, they ‘win over’ universals^29^, and we no more have to do with the relation as a universal but with an instance of it. But if a relation is not a universal, it cannot be a value, since values are universals. Can we, then, still insist that some value is responsible for the special, unique valuableness of the painting?

The answer is yes. For what may account for the special or unique value of the Mona Lisa is not necessarily Leonardo’s putative personal-particular value, but his ability to create outstandingly valuable artifacts. This is an ability that may be human-specific, but general, a true universal, which can be called ‘being able to create valuable things.’ But it must be added that we also have an ability to create disvalues, and to destroy values. Human beings routinely create values and disvalues, or rather, value/disvalue instances as they make or cause value/disvalue properties and relations to be instantiated^30^. The ability to do so is, in contrast to natural causation, itself a value/disvalue. It is partly responsible for the dis/valuableness as well as the worth of a particular. It is a relational property, and strictly speaking, it must be considered a relation, and rendered as ‘creates (dis)value’ (*a* creates [dis]value *X*, where *a* is a particular and *X* is a universal). Thus, it is one of the values possessed by the Mona Lisa that it was painted by someone who had the ability to create such valuable particulars.

But is particularity not completely lost? After all, we attribute great significance to the fact that the Mona Lisa was painted by Leonardo and not by any able individual. That is indeed true but there are two further things to be taken into account. First, perhaps paradoxically, the special, original value of the Mona Lisa is a matter of Leonardo’s having created other outstanding artifacts as well. His ability, or rather performance, of having created things that are unique in the sense analysed above (first interpretation) is affected by those things themselves, making *his worth* greater than the worth of an inferior artist (but presumably not greater than *all* other artists!). We may say with no more right that particularity is lost than that it has just been created.

And secondly, uniqueness in this sense should not be absolutized. Even if the Mona Lisa is unrepeatable, just because it was painted by a great artist who died long ago, it does not represent a value that cannot be compared to anything else^31^. It is valuable partly because it was painted by Leonardo, but that is only part of its worth. We make comparions between ‘unique’ things routinely and express the result, among other ways, in our willingness to buy them at a given price, or to sacrifice other valuable things in their favor. There are many outstanding painters, for instance, whose works are in one sense incomparable (uniqueness), but in another sense quite comparable (and expressible in terms of prices).

Thus, the objection to the view that values are universals taken from the fact that we often cherish and value things because of their particularity, uniqueness, unrepeatability, hence not all values are universals, can be countered most effectively by realizing the value inherent in the human ability of creating value-instances. From this the worth of particular individuals (in this example, of a painter) can be deduced which then contributes to the instantiation of the supposed special or unique worth of certain particulars. This explanation is consistent with the conception of value instantiation outlined here.

## Rarity

As a final objection, let us consider the supposed value of rarity. For it is obvious that there are objects considered to be valuable by many people exclusively on account of their rarity but not of any value they bear, nor of the worth of the particulars they belong to. Evidently, a rare stamp or a precious pearl cannot be connected axiologically to the value-creating ability of their makers. Whereas it might be impossible to duplicate a rare stamp because the particular state of affairs in which it was produced has already ceased to exist^32^, no such problem arises for a pearl, except the time that is needed for it to be formed. Of course, many rare objects do bear certain values, but quite often these are merely of additional nature (the stamp may not be beautiful at all, though aesthetic qualities may increase its price).

But it is also pretty obvious that simple rarity is insufficient to explain the value of such things. At the extreme, any particular may be considered rare, or rather, unique, from which no value arises (see the previous section). And there are many unique objects in the universe that have no or only very little such value (e.g. any pebble or snowflake), or are positively disvaluable (perhaps the prototype of new lethal weapon, or a meteorite threatening the Earth). Taken ‘rarity’ less extremely, and allowing for groups of objects that are considered to be ‘rare,’ that is, not unique but ‘small in number’ (another relation), we see that rarity such understood is still in itself not a value^33^. It is, however, equally obvious that the unique or particular value of a rare stamp has to do something with its being rare, its being a stamp, and its being collected (sought for).

For a piece of paper to qualify as a stamp, it must satisfy a number of requirements, among which certain properties and, perhaps more essentially, relations figure. For instance, it must be ‘issued by a sovereign authority’ (which is an internal relation). And it must ‘be (have been) capable of being used in normal mailing.’ But these qualities of stamps do not make them valuable. Since rarity in itself is not a value, we should look for other candidates.

Stamps collectors usually have a very special attitude towards stamps, which is similar to, but also different from, the attitude collectors of other curious objects have towards their respective objects. The roots of such attitudes can be rather different, but usually and originally linked to some values. In the case of stamps, aesthetic values prefigure. In the case of relics, religious values come to mind. In the case of autograms, value-creating abilities of artists lie in the background^34^. These values might become rather blurred, and, as was said above, in particular cases they really are only of additional nature. The age when relics of saints were passionately, often violently, collected, is over, though the number of relics has not significantly increased in the meantime. Autograms of pop stars that were in great demand long ago are valueless today.

These values vanishing, other value-grounds are necessary to explain the basic relation collectors have to their cherished objects, whatever they actually are. Since the relation ‘collects’ does not have any value-aspect in itself, and there is no other verb to make the required distinction, let us simply use an asterisk to denote the relation value ‘Collects*^35^.’ This is similar to value-creation, as it expresses a special human ability, no less deeply rooted in human nature than value-creation, but having a less intense or robust value-aspect^36^. Most probably, there is a special joy or enjoyment arising out of it. Even those who do not collect stamps may appreciate the enjoyment of stamp collectors. Thus, although the objects (particulars) human beings like to collect* may have some connections to values, this in itself is not sufficient to instantiate a new value on which the total worth of a rare, collected* object supervenes. But we have the testimony of times that human beings simply like to collect* various things and this is the universal relation having a certain value-aspect the instantiation of which explains the axiological fact that certain objects seem to be valuable in virtue of their rarity. Since it is also a well-known fact that whether stamps or certain photos or telephone cards are being collected* depends not only their sheer rarity, but also on whether or not they are in fashion, therefore ‘being in fashion’ (which is another relation, or combination of relations) decisively influences the context in which the relation of collects* obtains and hence contributes to the instantiation of the value relation of ‘Collects*^37^.’

It seems, then, that to explain the value customarily attributed to rarity we can draw on another universal, the relation value of Collects* even though in most cases of collected* objects certain other value universals also play a role. In contrast to the relation of ‘Creates value-instances’ the relation of ‘Collects*’ instantiates the property of being a value to a lesser extent, and in its instantiation the worth of the relata is of lesser importance, compared to the importance of the context (how many people, how ardently seeking the rare objects).

## Conclusion

Axiologists have had hard time in explaining Moorean wholes which can have different value indexes than their components. In this paper I proposed to use a realist ontological framework in which instantiation of universals is crucial to explaining how the world exists. It was claimed that in a realist conception values are universals, both properties and relations. Instantiation is an operation: how are universals linked to particulars? But the usual understanding of this problem is static. It is preoccupied with the ‘link,’ *its* nature, *its* function, *its* belonging to this or that category of entities. It was suggested here that instantiation is also a dynamic problem. The question of „how are universals linked to particulars?” is to be understood also as „in virtue of what is a universal instantiated?” A number of value relations were then discussed and it was argued that a pluralistic conception is the best answer. The content of the relation, the relata, and the context (the instantiation of further relations) may all be responsible for the instantiation of the relation value. The other important result was that there is a further property, the one of being a value which is a higher-order property which can be instantiated by relation (and property) values to various degrees. This also plays a decisive role in the instantiation of individual values.

The main lesson of this approach for axiology is that if values are universals, which also implies value pluralism, then the problem of organic or complex wholes having a value which is not identical to the sum total of the values of its parts can be explained, rather than merely asserted. From this it also follows that if these conclusions are sound, and the explanatory gain real, then the axiological approach which starts out from a moderate realist ontology is also vindicated.

Finally, a few hints at the possible consequences of this approach to the theory of intrinsic value may be in order. It was argued earlier that the problem of ‘value bearing’ itself suggests a realist approach. However, the various proposals about ‘what’ bears value indicate that to most axiologists ‘value bearing’ is tantamount to ‘intrinsic value bearing.’ The notion of ‘intrinsicality’ is by no means crystal clear, F. Feldman distinguishes between eight different meanings^38^. What is clear, however, is that this notion is meant to connect axiology to ethics, in the Moorean tradition. The present account remains a step backwards as has been emphasized several times. It is not the ethical intuitions that need to be founded in axiology. Rather, it is the axiological intuitions or, to be more accurate, the axiological experiences, our encounters with values that have to be accounted for first. There are many different encounters that present us with the world of many distinct values. Since they are all values, we do need to account for this fact as well, hence the conclusion that there is such a property as being a value. In this context intrinsicality may have two meanings. First, in a trivial sense, all values *qua universals* are intrinsic (and disvalues are qua universals also intrinsic). Of course, all relations and properties are intrinsic in this, rather vacuous, sense where intrinsicality looks very much like identity. Secondly, values *qua values* (that is, participating in the higher order universal of being value) are also intrinsic. Intrinsicality is here understood as a logico-existential requirement of any value to be a value: any value must participate in the property of being value in order to qualify as a value.

## Endnotes

^1^ Sometimes we find a distinction made between intrinsic goodness and intrinsic badness, e.g. in Zimmerman (2001) usually attached to states (of affairs) or facts, but value and goodness are probably distinct categories and the concept of intrinsic badness might not be identical with intrinsic disvalue.

^2^ For suggestive attempts to explain instantiation see Mertz (1996) and Baxter (2001). Both authors use the term ‘aspect’ but their explanations are different.

^3^ ‘Being a spoon’ is a property that hardly can be instantiated only partially or ‘to some extent.’ But ‘being money’ is a property that is instantiable to different degrees. Economists may disagree on what financial instruments are to be counted as ‘money.’

^4^ For a view that values are born by physical things and persons see Rabinowicz-Rønnow-Rasmussen (2001); concrete things: Tännsjö (2005); concrete states of individuals: Chisholm (2005); experiences: Audi (2003); states of affairs: Lemos (1994); facts: Zimmermann (2001).

^5^ Moore (1988). His treatment of the topic is responsible for this. His concern was to redirect ethical theory and he uses the term ‘intrinsic value’ to get to the notion of the ‘good’ as soon as possible. Thus, the very first occasion to employ the term ‘intrinsic value’ or – as he writes – ‘intrinsic worth’ is embedded in a longer discussion of the ‘good’ as ‘the unique property of things’ (9., 17). There is no room for a consideration of individual values in this context.

^6^ ‘Love’ is the content of the relation ‘loves.’ For grammatical simplicity, the content will be identified with the relation itself, unless the context requires stressing the distinction.

^7^ I assume that love between persons (and possibly collective subjects) is substantially different from love that connects a person and an object or concept. („[T]he proper, natural object of love is a person.” Mulligan (1998), 173.

^8^ Note that the virtual presence of values is an epistemological, and not an ontological, statement. Values and disvalues influencing the worth or valuableness of the particulars must be actually instantiated, otherwise the differences between the two cases could not be accounted for. This is why M. Zimmerman’s proposal, to explain the difference between right and wrong pleasure (pleasure at the good and pleasure at the bad) in terms of virtual and actual value is unconvincing. His distinction is ontologically ungrounded, as it remains unexplained what makes actual value turn virtual and vice versa. See Zimmerman (1999).

^9^ Another example could be the mutual supervenience of being a human being and being conscious of being a human being.

^10^ It seems possible to argue that, contrary to Aristotle’s suggestion, recklessness is not an extreme form of courage, but, at least in certain cases, it is a curious amalgam of courage and cowardice. For recklessness is a more or less conscious disregard for the consequences of one’s action, and this disregard may arise from a fear of having to face those consequences.

^11^ Lemos (1994), 46. Though not citing him, Irwin Goldstein makes the same point: „ being maliciously pleased has two components. The parts need not inherit the whole’s properties. Malicious pleasure’s moral offensiveness is a property of the whole – the cognitive-pleasure compound. We are not entitled to infer from the whole’s being bad and offensive that the pleasure component is also bad and offensive.” Goldstein, (2003), 28. This may be the case, indeed, but we still may wish to know why the whole is ‘bad;’ whether it is worse just because it contains a valuable component and if yes why, and so on.

^12^ The reason is that ‘Enjoys’ is evidently a relation, whereas ‘being Pleased at’ is less evidently a relation. Indeed, it is difficult to imagine cases where one is pleased in general, and not *at* something concrete, or is not pleased *by* something concrete, and feels pleasure like feeling distressed or sleepy, but such cases may, on an exceptional basis, be possible. Pain is similar. We are pained by something, or feel pain at something, but are rarely in pain not related to a cause. However, such cases might be possible. Difficult cases, favored by existentialism, include being in fear, being worried, etc., without any apparent reason.

^13^ This is the position Lemos endorses (1994, 40–46).

^14^ Olson (2004) also refers to the „intentional relation” of the relatum – the individual – as being constitutive of value. Further, he argues that the phenomenological identity of two experiences (both being pleasures) do not compel one to the contention that they are evaluatively also identical (that is, values). This latter possibility is, in my view, not fully explored. It is possible that the right moral evaluation of an experience of malicious pleasure makes that experience phenomenologically (and perhaps also psychologically) different from an experience of morally right pleasure.

^15^ A computer cannot enjoy anything. A person cannot enjoy her own consciouslessness or a future state of her that she is unaware of (such as, for instance, enjoying a birthday gift).

^16^ On valuing retributive punishment, see also Harman (2000), Chapter 8.

^17^ Since envy is a disvalue which can be analysed psychologically in terms of pain and pleasure (feeling pain at someone’s deserved pleasure) but it is not a combination of a value and a disvalue, we might conclude that, inversely, pleasure at another person’s pain must be an ontologically independent disvalue, even though there is no distinct word for it.

^18^ Olson (2004) talks about the „intentional relation” of the person feeling pain/pleasure at another person’s pain which is similar to my reference to the constitutive role of the relata in the instantiation of values.

^19^ Like suffering under envy, suffering under compassion is not direct physical pain.

^20^ No wonder that for many ethical theorists of the British moral philosophical tradition compassion, empathy or sympathy were even more fundamental phenomena than love.

^21^ Thomas Hurka claims, following Moore, that „ [i]f *x* is intrinsically evil, hating *x* for itself, though intrinsically good, is not as intrinsically good as *x* is intrinsically evil. Hurka (1992). The principle is perhaps just another way of expressing the axiological truth that relation values inherently connected with disvalues are always dependent on the obtaining of other, non-disvalue-related, relation values. But if the principle aims at a more precise guiding of how to *calculate* valuableness and disvaluableness (and worth), a more rigorously elaborated calculus is needed to assess the validity of the principle.

^22^ Matthew Pianalto challenges the view that pleasure is an intrinsic value. His argument is based on the distinction between intrinsic and instrumental value and is meant to show that pleasure is inherently instrumental to well-being, ‘the’ intrinsic good. But the argument may be adapted to the present conception as contending that pleasure is not identical with the higher-order property of being a value (in fact, no first-order property or relation can be identical with it), and pleasure may instantiate the property of being a disvalue, too. In any case, that pleasure is the highest or sole (intrinsic) value is a dubious claim. Pianalto (2009).

^23^ Another peculiar example is „ being a parent” (and/or „being a father/mother”). The interesting question is not whether parental (paternal, maternal) love is a value since love is a value, but whether being a father/mother, for instance, is a value not reducible to other values, including love, even if in practice parental *love* is, perhaps, inseparable from the consciousness, pride, and power of parenthood.

^24^ Catholic and Orthodox theology has subtle distinctions between reverence, adoration, worship etc.

^25^ Lady Diana’s dress is another object whose axiological status has been extensively discussed in the literature. To talk about its ‘holiness,’ in a however secular sense, sounds rather sacrilegious, but not necessarily to all modern ears. See Rabinowicz, Rønnow-Rasmussen (1999).

^26^ Axiology is, to repeat, not moral theory. Axiological results must influence moral reasoning but do not replace it. How to determine the worth of a person is a matter of moral theory.

^27^ A similar reasoning is possible for disvalues. It is sometimes suggested the there are cases of rank evil (persons like Stalin, events like the Holocaust) that are somehow „ more unique” than similar cases and this special status is partly explained in terms of a unique combination (though in principle repeatable) of disvalues.

^28^ Ch. Grau uses the same example, making the same point about the particular history that an original piece has to its author. (Grau, 2006). His own solution to the puzzle of irreplaceability being a value for its own sake, but not an intrinsic value (insofar as intrinsic value supervenes on the object’s intrinsic properties which are naturally repeatable) is to subsume it under the category of „final values” in the Korsgaardian sense, though in a distinct way. That is, his thesis is that uniqueness or particularity is a kind of value. What remains unclear is why and how not everything that exists (the universe being made up of particulars) appears to have the *same* value in virtue of its particularity.

^29^ Armstrong (1978).

^30^ Value creation is not to be understood as creating universals but as making them obtain.

^31^ The comparability of values is a topic beyond the scope of this paper. The suggestion here is that even if particularity in itself were a value, it would still be only one distinct value, contributing to the valuableness or worth of the particular in question only partially, and not at all precluding evaluative comparisons with other particulars.

^32^ Of course, the latter case is more doubtful, since technology allows for more and more chances to restore a former technological state of affairs.

^33^ John O’Neill, who considers the problem of rarity with view of environmental ethics, writes that „[r]arity appears to confer a special value to an object. This value is related to that of another irreducibly relational property of environmental significance, i.e., diversity” (124). This claim is related to another one, that the existence of values does not presuppose the existence of human beings (evaluators). The latter claim is consistent with the present approach but the former is not. Rarity seems to be constitutive of a relation value specific to human beings (see below) but not, in itself, a value. Environmental ethics is supportable on the grounds that some, but not all, values are objective in the sense that no conscious evaluators are necessary for them to exist. Further, diversity (variety) as a value is ambiguous. “It” might take up a value and a disvalue aspect as well, depending on the context. A single rubin stone on a dress might be more refined and elegant than a variety of precious stones. Variety might be related to opulence, confusion, scare, even disgust. See O’Neill (1992).

^34^ There are even more dubious cases, such as four-leaf clovers which are, indeed, rare, and thought to be valuable by some people, but considered absolutely worthless by most people.

^35^ Collecting* is different from simply collecting insofar as the former relation has an aspect of enjoyment, pride, mastering, and especially treasuring, etc. that have clear value-connections. This is perhaps what Christine Korsgaard had in mind when considering „ [m]ink coats and handsome china and gorgeously enamelled frying pans” and saying that they “are all things that human beings might choose partly for their own sakes under the condition of their instrumentality: that is, *given the role such things play in our lives*” (Korsgaard 2005, 89, emphasis added). Of course, her Kantian conception of value relates every value to humanity, which is questionable, but relation values, as was said earlier, might indeed turn out to be essentially linked up with the existence human beings (though enthusiasts about animals may disagree, saying that parental love, for instance, is a relation value instantiable in many species). Zimmerman makes a similar point: “ethical goodness is relative (…) to persons” (*The Nature of Intrinsic Value,* 27).

^36^ That this might not be a value relation is a view that J. Swift appears to endorse. In Gulliver’s Fourth Travel, Ch. 7., we find a description of the detestable yahoos’ passion for collecting and hiding worthless stones.

^37^ The reasons of why certain things are in fashion are famously difficult to explain. Historically, one of the most amusing example is the tulipomania in the 16th century Holland.

^38^ Feldman (1998).
